# Dynamic Phycobilin Pigment Variations in Diazotrophic and Non-diazotrophic Cyanobacteria Batch Cultures Under Different Initial Nitrogen Concentrations

**DOI:** 10.3389/fmicb.2022.850997

**Published:** 2022-06-02

**Authors:** Jingyu Wang, Nicole D. Wagner, James M. Fulton, J. Thad Scott

**Affiliations:** ^1^The Institute of Ecological, Earth & Environmental Sciences, Baylor University, Waco, TX, United States; ^2^Center for Reservoir and Aquatic Systems Research, Baylor University, Waco, TX, United States; ^3^Department of Geosciences, Baylor University, Waco, TX, United States; ^4^Department of Biology, Baylor University, Waco, TX, United States

**Keywords:** cyanobacteria blooms, stoichiometry, eco-physiological traits, nutrient limitation, phycobilin pigment

## Abstract

Increased anthropogenic nutrient loading has led to eutrophication of aquatic ecosystems, which is the major cause of harmful cyanobacteria blooms. Element stoichiometry of cyanobacteria bloom is subject to nutrient availabilities and may significantly contribute to primary production and biogeochemical cycling. Phycobilisome is the antenna of the photosynthetic pigment apparatus in cyanobacteria, which contains phycobilin pigments (PBPs) and linker proteins. This nitrogen (N)-rich protein complex has the potential to support growth as a N-storage site and may play a major role in the variability of cyanobacteria N stoichiometry. However, the regulation of PBPs during bloom formation remains unclear. We investigated the temporal variation of N allocation into PBPs and element stoichiometry for two ubiquitous cyanobacteria species, *Microcystis aeruginosa* and *Dolichospermum flos-aquae*, in a batch culture experiment with different initial N availabilities. Our results indicated that the N allocation into PBPs is species-dependent and tightly regulated by the availability of nutrients fueling population expansion. During the batch culture experiment, different nutrient uptake rates led to distinct stoichiometric imbalances of N and phosphorus (P), which substantially altered cyanobacteria C: N and C: P stoichiometry. *Microcystis* invested cellular N into PBPs and exhibited greater flexibility in C: N and C: P stoichiometry than *D. flos-aquae*. The dynamics of such N-rich macromolecules may help explain the N stoichiometry variation during a bloom and the interspecific difference between *M. aeruginosa* and *D. flos-aquae*. Our study provides a quantitative understanding of the elemental stoichiometry and the regulation of PBPs for non-diazotrophic and diazotrophic cyanobacteria blooms.

## Introduction

The global rise of cyanobacteria blooms has threatened ecosystem and human health (Paerl and Otten, 2013). Therefore, understanding the mechanisms controlling cyanobacteria proliferation is of utmost importance (Huisman et al., 2018). Cyanobacteria flourish when environmental conditions are favorable, but blooms can draw down the available nutrient pool quickly (Davis et al., 2010; Chaffin et al., 2013). Changes in nutrient availability alter the elemental stoichiometry of cyanobacteria, which is known to have a central effect on primary production and biogeochemical cycling (Paerl and Pinckney, 1996). However, there is a lack of knowledge about cyanobacterial growth dynamics and stoichiometric variation during bloom formation. Although a wealth of continuous culture studies is available in the literature that examines the effects of changing nutrient supply on phytoplankton stoichiometry and growth physiology (see references in Hillebrand et al., 2013), they may not reflect the complexity of the aquatic ecosystem because resource availabilities are rare at equilibrium in natural settings.

Besides nutrient condition, another potential constraint of elemental stoichiometry is the growth rate. Fast-growing organisms usually require a high concentration of ribosomes, which are phosphorus (P)-rich macromolecules, providing a fundamental biochemical basis for the widely accepted growth rate hypothesis (GRH, Elser et al., 2000). Therefore, the GRH is frequently used to predict a decline in biomass C: P ratio with increasing growth rate and there is evidence supporting such prediction (Liu et al., 1999; Kruskopf and Flynn, 2006; Dick et al., 2021). In addition, a meta-analysis found phytoplankton exhibit more constrained stoichiometry at higher growth rates (Hillebrand et al., 2013). However, most systematic approaches to understanding mechanisms that regulate elemental stoichiometry have focused on eukaryotic phytoplankton, despite the substantial influence of cyanobacteria on nutrient cycling in freshwater systems (Cottingham et al., 2015).

The elemental stoichiometry of cyanobacteria largely depends on the relative abundance of a handful of macromolecules, such as proteins, carbohydrates, lipids, nucleic acids, and pigments (Geider and La Roche, 2002). Among those molecules, phycobilin pigments (PBPs) are N-rich pigments with the ability to capture light across a broad spectral range (Croce and van Amerongen, 2014). Besides light-harvesting, PBPs degrade in response to nitrogen (N) limitation, to avoid photo-damage and the phycobilin protein complex degrades to re-allocate amino acids to maintain vital cellular activities (Schwarz and Forchhammer, 2005). The phycobilisome complex contains the PBPs with other polypeptides that occur with a strict stoichiometry of pigment chromophores to proteins within the phycobilisome (Tandeau de Marsac, 2003; Stadnichuk et al., 2015). PBP concentrations are highly sensitive to both light and N availability (Wang et al., 2021), indicating that the variability in such N-rich macromolecules may provide a physiological basis for elucidating the interaction between the growth of cyanobacteria and aquatic ecosystem N stoichiometry. Although PBP concentrations were regulated differently in diazotrophs than non-diazotrophs when blooms were driven to N limitation or sufficiency (Wang et al., 2021), our previous work did not measure PBP temporal variation caused by changing resource concentrations.

*Microcystis* and *Dolichospermum* are among the most ubiquitous cyanobacterial genera globally and are often the cause of harmful algal blooms (HABs, Harke et al., 2016; Li et al., 2016). Although they share some physiological traits that help them proliferate in freshwater ecosystems (Carey et al., 2012), they exhibit different mechanisms to cope with N limitation. N_2_ fixation presumably improves the fitness of *Dolichospermum* under N limitation (Tilman et al., 1982); however, *Microcystis* was shown to effectively compete with N_2_-fixing cyanobacteria under conditions that are favorable for diazotrophic growth (Paerl et al., 2014). Interestingly, phycobilisome and PBP degradation may be part of a long-term survival strategy for non-diazotrophic cyanobacteria because of their potential as N-storage site in the cell (Schwarz and Forchhammer, 2005). Compared with cyanophycin, which has also been shown to act as an N-reservoir molecule (Flores et al., 2019), PBPs contain less N but are more abundant in cyanobacteria cells, with a PBPs: biomass carbon mass ratio up to 0.4 (Wang et al., 2021). Thus, PBPs may be a prominent intracellular N pool in cyanobacteria.

It is well-established that cyanobacteria growth rate varies based on ambient nutrient concentrations, which can alter elemental stoichiometry. However, studies that quantify and compare the correlations between growth rate, stoichiometry, and environmental nutrient concentrations in batch cultures are limited, particularly for *M. aeruginosa* and *D. flos-aquae*. The batch culture approach is urgently needed to better understand how a fast population expansion affects ambient nutrient concentration, population biomass dynamics, and elemental stoichiometry of known HAB species. The aim of this study was to investigate how non-diazotrophic and diazotrophic cyanobacteria modulate their elemental stoichiometry and PBP metabolism following population grow and nutrient depletion in batch cultures. We hypothesized that PBPs are dynamically synthesized and metabolized differently by non-diazotrophs (*M. aeruginosa*) and diazotrophs (*D. flos-aquae*) according to instantaneous dissolved nutrient availability that varies as population increase in a batch culture. Following population increase, both species are proposed to modulate elemental stoichiometry as ambient nutrient concentrations become scarce, but non-diazotrophs are predicted to be more flexible in nutrient stoichiometry than diazotrophs.

## Materials and Methods

### Cultures, Culture Maintenance, and Growth Conditions

The unicellular non-diazotrophic cyanobacterium *Microcystis aeruginosa* strain 2385 and the filamentous diazotrophic cyanobacterium *Dolichospermum flos-aquae* strain 1444 were obtained from the culture collection of algae at the University of Texas at Austin (UTEX). Cultures were maintained on sterile 0.5× BG11 medium (Sigma C3601). Batch cultures were grown in Erlenmeyer flasks at 26 °C on a 14-h: 10-h light: dark cycle and irradiance of ∼ 100 μmol/m^2^/s measured by a quantum meter (Spectrum Technologies, 3415FQF). Cultures were maintained by transferring 1% cell culture into freshly prepared medium monthly.

### Effect of N Availability on Stoichiometry and Phycobilin Pigments Metabolism

To examine the effect of inorganic N pool size on PBP dynamics, quadruplicate experimental units were made by combining 5% N-free BG-11 (357 μg/L phosphorus) with 1.35 μg/L vitamin B_12_ and manipulating N concentrations (322, 2,576, and 16,128 μg/L as nitrate N for low-N, intermediate-N, and high-N treatment, respectively). The initial cell densities to start the experiments were ∼ 9.0 × 10^8^ and 1.0 × 10^9^ cells/L for *M. aeruginosa* and *D. flos-aquae*, respectively. Cyanobacteria were cultivated on a 14-h:10-h light: dark cycle with constant temperature of 26°C and light intensity of 100 μmol/m^2^/s measured by a Quantum meter (Spectrum Technologies, 3415FQF). We used batch cultures to simulate the rapid population expansion and associated nutrient decline of natural cyanobacterial blooms. Growth was monitored by measuring *in vivo* chlorophyll *a* fluorescence (RFU; Turner Designs Trilogy) during the experiment. In addition, 2 mL sub-samples were preserved with Lugol’s iodine daily for cell enumeration. Sampling was scheduled to examine N stoichiometry and PBP concentrations following population expansion (for temporal RFU variations, see Supplementary Figure 1). On Days 5, 7, 9, 11, 12, and 14, cells were harvested by filtering onto 0.7 μm pre-combusted GF/F glass fiber filters to determine particulate C/N, particulate P, and PBPs. Filters were stored at −20°C until analyzed. In addition, filtrate samples were saved and stored at −20°C on each sampling day for nitrate-N and soluble reactive P (SRP) analysis.

### Determination of Particulate C, N, P, and Cell Enumeration

First, particulate C and N samples were analyzed by drying the filters at 60°C for 24 h. After drying, filters were analyzed using an elemental analyzer (Thermo-Fisher Flashsmart NC soil, CE Elantech, United States). Particulate P was determined using the molybdate blue colorimetric method (American Public Health Association [APHA], 2005). Briefly, filters were first digested in persulfate and read on a UV–visible spectrophotometer based on the molybdenum blue method at 885 nm.

We used cell concentration to calculate the specific growth rate (μ) from the following equation:


(1) $$\mu =\frac{l\,n\,\left(\frac{C_{2}}{C_{1}}\right)}{t_{2}-t_{1}}$$

where C_2_ and C_1_ are the cell concentrations on Day t_2_ and Day t_1_, respectively.

For *M. aeruginosa*, cell concentrations were determined using a flow cytometer (BD Diagnostic Systems, FACSVerse, San Jose, CA, United States) with forward-scatter side-scatter method as previously described by Wagner et al. (2019). Quality control was performed using standardized beads to check for instrument functionality. For *D. flos-aquae*, cell counts were performed using a microscope at 400× magnification (Nikon Eclipse 80i, Japan) on a subset of samples and cell counts were related to *in vivo* chlorophyll *a* fluorescence (*R*^2^ = 0.94, *p* < 0.001, degrees of freedom = 26, Supplementary Figure 2). The obtained linear equation was applied to *in vivo* chlorophyll *a* data for samples without direct cell counts to predict cell concentration.

### Determination of Phycobilin Pigment, Nitrate-N, and Soluble Reactive P Concentrations

Phycobilin pigment extraction was conducted according to Khazi et al. (2018) with minor modifications. Briefly, PBPs from cyanobacteria captured onto filters were extracted by adding 5 mL of 0.1 M phosphate buffer (pH 7.0) into centrifuge tubes. After shaking, the cell suspension was then stored at 4°C for 12 h. The slurry was then sonicated at 35 k Hz for 2 min (VWR ultrasonic cleaner) and then centrifuged at 4500 *g* for 5 min (Thermo Scientific, United States) at 4°C. The resulting supernatant was used for the determination of phycocyanin, allophycocyanin, and phycoerythrin on an UV–Vis spectrophotometer (Beckman, United States) with 1-cm cuvette, according to Bennett and Bogorad (1973). Phycocyanin (PC), allophycocyanin (APC), and phycoerythrin (PE) concentrations were calculated based on the following equations:


(2) $$P\,C\,\left(m\,g/L\right)=\frac{1000\times \left(A_{615}-0.474\times A_{652}\right)}{5.34}$$

(3) $$A\,P\,C\,\left(m\,g/L\right)=\frac{1000\times \left(A_{652}-0.208\times A_{615}\right)}{5.09}$$

(4) $$P\,E\,\left(m\,g/L\right)=\frac{1000\times \left(A_{562}-2.41\times P\,C-0.849\times A\,P\,C\right)}{9.62}$$

(5) T⁢o⁢t⁢a⁢l⁢P⁢B⁢P⁢s⁢(m⁢g/L)=P⁢C+A⁢P⁢C+P⁢E

where A_615_ is the absorption at 615 nm, A_652_ is the absorption at 652 nm, and A_562_ is the absorption at 562 nm.

Due to the strict stoichiometry of pigment chromophores to linker proteins within the phycobilisome, we used the PBP concentration as a proxy for the protein complex. The amount of N in each PBP was estimated based on their chemical composition (phycocyanin: C_33_H_38_N_4_O_6_, allophycocyanin: C_33_H_42_N_4_O_6_, phycoerythrin: C_33_H_38_N_4_O_6_; Stadnichuk et al., 2015); thus, N allocated to PBP (PBP-N) per cell was calculated.

Dissolved nitrate N and SRP were analyzed using a Lachat 8500 flow-injection auto-analyzer with an ASX-520 autosampler (Hach Co., Loveland, CO, United States) according to EPA QA/QC standards and APHA/CRASR protocols (American Public Health Association [APHA], 2005); Center for Reservoir and Aquatic Systems Research, Waco, TX, United States).

### Statistical Analysis

We examined how ambient nitrate-N and SRP concentrations affected PBP-N cell quota by fitting Michaelis–Menten equation, linear equation, or piecewise linear equation to the data. Regression models were selected based on the Akaike information criterion (AIC). The same number of observations was used for each model compared by AIC. Similarly, we examined the relationship between ambient N: P ratio and cyanobacteria C: N, C: P ratios using linear regression or piecewise linear regression. Although growth rate, ambient nutrient concentration, and cyanobacteria stoichiometry are colinear, our focus was to compare the relationships between the two species and was less concerned about the predictive power of our regressions. We examined the relationship between cyanobacteria growth rate and stoichiometry, PBP cell quota using a linear regression. In addition, we tested whether the linear regression slopes in these analyses differed between *Microcystis* and *Dolichospermum* populations using the standardized major axis (SMA) analysis by Standardized Major Axis Tests and Routines software (SMATR, Wright et al., 2006) that calculates the slope of a regression with the corresponding confidence intervals that can be used to determine whether two regression lines have the same slope. All regressions were performed with R software (version 3.5.3), and data visualization was done by ggplot2 (Wickham, 2016).

## Results

### Temporal Variations of Cyanobacteria Population and Dissolved Nutrients During Batch Culture Experiment

**FIGURE 1:**
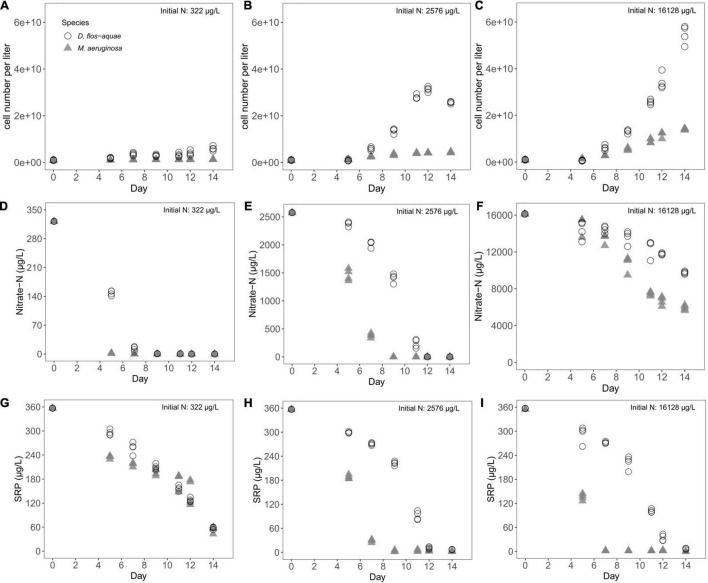
Temporal variations of cell concentration **(A–C)**, nitrate-N concentration **(D–F)**, and SRP concentration **(G–I)** during a batch culture experiment with different initial N concentrations.

Both *M. aeruginosa* and *D. flos-aquae* populations expanded proportionally to the initial N concentrations of the experiment reaching maximum biomass after 12–14 days. The *M. aeruginosa* populations reached maximum biomass of 1.3 × 10^9^ cells/L in 322 μg/L initial N (Figure 1A), 4.3 × 10^9^ cells/L in 2,576 μg/L initial N (Figure 1B), and 1.4 × 10^10^ cells/L in 16,128 μg/L initial N (Figure 1C). The *D. flos-aquae* populations reached maximum biomass of 6.0 × 10^9^ cells/L in 322 μg/L initial N (Figure 1A), 2.6 × 10^10^ cells/L in 2,576 μg/L initial N (Figure 1B), and 5.5 × 10^10^ cells/L in 16,128 μg/L initial N (Figure 1C).

Expansion of both cyanobacteria populations corresponded with a decrease in dissolved nutrient concentrations across all treatments. Nitrate-N concentration dropped from 322 μg/L to less than 3 μg/L on Day 5 in *M. aeruginosa* cultures, but the nitrate-N pool was not exhausted for *D. flos-aquae* until Day 9 (Figure 1D). Under 2,576 μg/L initial N condition, nitrate-N concentration was less than 3 μg/L on Day 9 for *M. aeruginosa*; however, *D. flos-aquae* did not completely consume nitrate-N pool until Day 12 (Figure 1E). Nitrate-N concentration only fell to ∼6,000 μg/L for *M. aeruginosa* and 9,700 μg/L for *D. flos-aquae* (Figure 1F) in cultures with the highest initial N conditions. Although the initial SRP concentration was same (357 μg/L), two species consumed SRP at different rates. The SRP concentration was ∼ 60 μg/L for *M. aeruginosa* and *D. flos-aquae* on Day 14 under 322 μg/L initial nitrate-N condition (Figure 1G). In contrast, SRP concentrations decreased to ∼3 μg/L on Day 9 for *M. aeruginosa* cultures and 7 μg/L on Day 14 for *D. flos-aquae* cultures when initial nitrate N was 2,576 μg/L (Figure 1H). With 16,128 μg/L initial nitrate-N concentration, the SRP pool was exhausted on Day 7 for *M. aeruginosa* cultures, while SRP concentration was ∼7 μg/L on Day 14 for *D. flos-aquae* cultures (Figure 1I).

### Effect of Nitrate-N Concentration on Cyanobacteria N Allocation to Phycobilin Pigment

**FIGURE 2:**
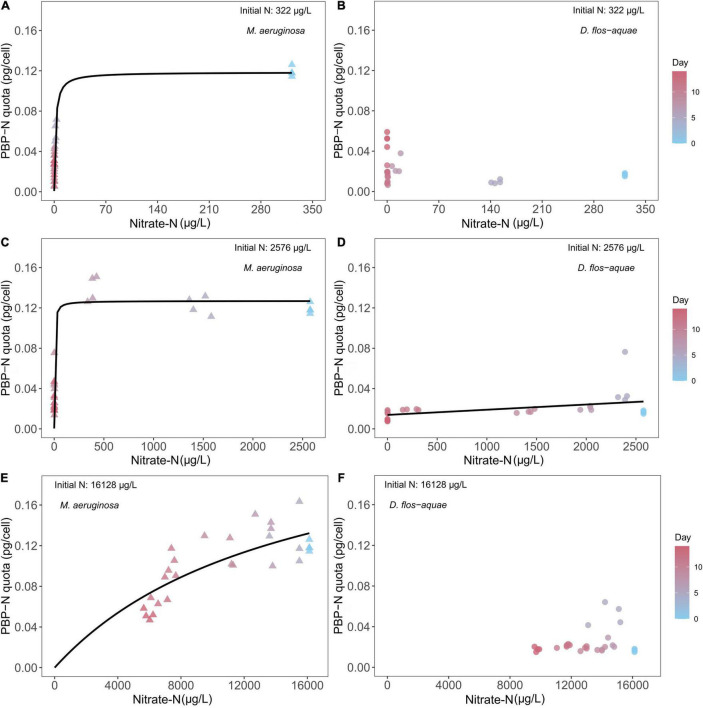
Relationship between cyanobacteria N allocated in PBP per cell (PBP-N quota) and ambient nitrate-N concentrations for *M. aeruginosa* **(A,C,E)** and *D. flos-aquae* **(B,D,F)** under different initial N concentrations.

The pattern of nitrate-N drawdown following population increase (Figure 1) allowed us to compare PBP dynamics against varied nitrate-N concentrations. It is important to note that this dynamic occurs because population expansion causes nitrate-N drawdown. When *M. aeruginosa* culture was supplied with 322 μg/L nitrate N initially, we found that the cell quota PBPs expressed as N (i.e., PBP-N) responded to nitrate-N concentrations in a Michaelis–Menten curve (Figure 2A), with a maximum PBP-N quota of 0.12 pg/cell [95% confidence range: (0.10, 0.13)] and a half-saturation concentration of 1.75 μg/L [95% confidence range: (1.10, 2.83)]. We fit a Michaelis–Menten function to these data even though some critical data were missed between 10 and 250 μg/L nitrate N. For *D. flos-aquae*, we observed that the PBP-N cell quota slightly increased by the end of the experiment (Figure 2B). However, no significant correlation was found between nitrate-N concentrations and PBP-N (linear regression, F = 2.12, *p* = 0.16, df = 26). Similarly, a Michaelis–Menten curve fitted for *M. aeruginosa* cultures under 2,576 μg/L nitrate N (Figure 2C) yielded a model that had a maximum PBP-N quota of 0.13 pg/cell [95% confidence range: (0.12, 0.14)] and a half-saturation concentration of 3.18 μg/L nitrate N [95% confidence range: (2.04, 5.37)]. However, for *D. flos-aquae*, the rate of change in PBP-N cell quota was constant during 14 days of batch culture experiment as we identified a significant linear regression between PBP-N cell quota and nitrate-N concentration (Figure 2D, Linear regression F = 7.48, *p* = 0.011, df = 26). Under 16,128 μg/L initial N conditions, we fit a Michaelis–Menten curve for *M. aeruginosa* with a maximum PBP-N quota of 0.25 pg/cell [95% confidence range: (0.18, 0.43)] and a half-saturation concentration of 14,820 μg/L nitrate N [95% confidence range: (7,585, 33,333); Figure 2E]. In contrast, PBP-N quota of *D. flos-aquae* populations appeared to be insensitive to nitrate-N concentrations (Figure 2F, Linear regression, F = 1.34, *p* = 0.26, df = 26).

### Effect of Soluble Reactive Phosphorus Concentration on Cyanobacteria N Allocation to Phycobilin Pigments

**FIGURE 3:**
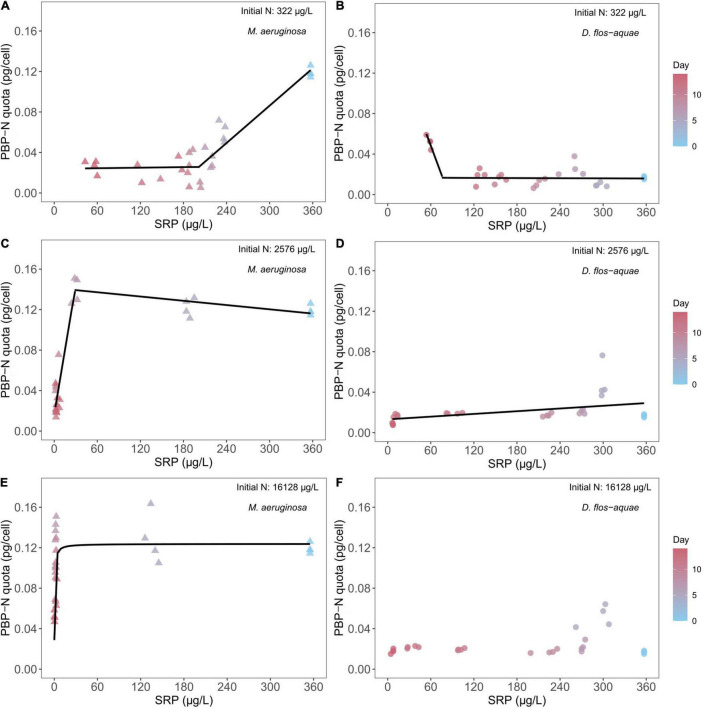
Relationship between cyanobacteria N allocated in PBP per cell (PBP-N quota) and ambient SRP concentrations for *M. aeruginosa* **(A,C,E)** and *D. flos-aquae* **(B,D,F)** under different initial N concentrations.

We found different correlations between SRP concentration and PBP-N cell quota during a batch culture experiment across different initial N conditions and between two species under a same initial N condition. For 322 μg/L initial N batch cultures, *M. aeruginosa* decreased its PBP-N cell quota from ∼0.12 pg/cell PBP-N to ∼ 0.04 pg/cell as population growth decreased SRP concentrations (Figure 3A). A piecewise linear regression identified a breakpoint of 201.5 μg/L SRP [95% confidence range: (139.4, 237.1)] that separated high and low rates of PBP-N decrease during the experiment (see slopes in Supplementary Table 1). Conversely, *D. flos-aquae* had a PBP-N quota of ∼0.02 pg/cell initially and maintained PBP-N cell quota at less than 0.04 pg/cell while SRP concentration decreased to ∼120 μg/L, after which PBP-N cell quota increased to ∼0.05 pg/cell (Figure 3B). Piecewise linear regression identified a breakpoint of 76.7 μg/L SRP [95% confidence range: (64.6, 167.1)] for *D. flos-aquae*. Under 2,576 μg/L initial N conditions, *M. aeruginosa* slightly increased PBP-N quota from Day 0 to Day 9 as SRP concentration decreased, then PBP-N quota declined to ∼0.03 pg/cell on Day 14 (Figure 3C). The breakpoint for SRP was estimated as 28.4 μg/L [95% confidence range: (16.8, 32.1)]. In contrast, *D. flos-aquae* regulated PBP-N cell quota according to SRP concentration with a constant rate as we found a significant linear regression (Supplementary Table 1 and Figure 3D). At the greatest initial N conditions, *M. aeruginosa* PBP-N cell quota responded to decreasing SRP concentration in a Michaelis–Menten curve (Figure 3E), with a maximum PBP-N quota of 0.12 pg/cell [95% confidence range: (0.11, 0.14)] and a half-saturation SRP concentration of 0.36 μg/L [95% confidence range: (0.16, 0.66)]. However, *D. flos-aquae* PBP-N cell quota was more constant during the experiment and insensitive to SRP concentration (Supplementary Table 1 and Figure 3F).

### Effect of Ambient Nitrate: Soluble Reactive Phosphorus Ratio on Cyanobacteria Stoichiometry

**FIGURE 4:**
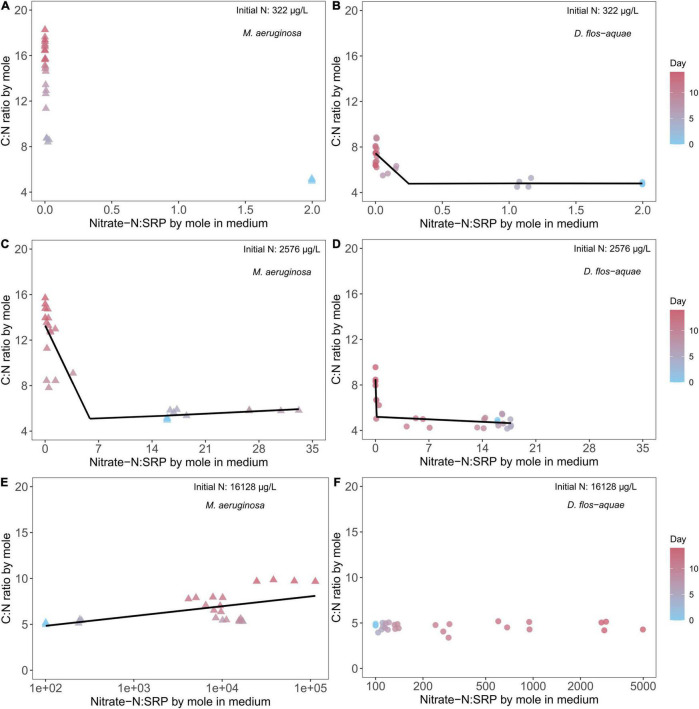
Relationship between cyanobacteria C: N ratio and ambient N: P ratio for *M. aeruginosa* **(A,C,E)** and *D. flos-aquae* **(B,D,F)** under different initial N concentrations.

The rates of nitrate-N and SRP drawdown during a population expansion resulted in variable ambient nitrate: SRP ratios over the course of batch culture experiment which resulted in unique patterns in C: N stoichiometry among species and across initial N conditions. The nitrate-N: SRP ratio (by mole) was 2 for the low-N (322 μg/L) treatment at the beginning of the experiment and decreased through time as nitrate N was exhausted and SRP remained saturated for *M. aeruginosa* populations. As a result, the ambient nitrate: SRP stayed below 0.1 from Days 5 to 14. Correspondingly, *M. aeruginosa* C: N ratio was close to 5 on Day 0 and increased to ∼17 on Day 14 (Figure 4A) while *D. flos-aquae* C: N ratio was less variable from 5 to 8 across the duration of the experiment (Figure 4B). Piecewise linear regression identified an ambient nitrate: SRP ratio of 0.24 [95% confidence range: (0.016, 0.47)] as the breakpoint for *D. flos-aquae* (Supplementary Table 2), which separated high and low rates of C: N change during the experiment (see slopes in Supplementary Table 2). The intermediate-N treatment (2,576 μg/L) created an initial nitrate-N: SRP ratio of 16, and this ratio increased to ∼30 on Day 7 and then decreased to less than 0.1 on Day 14 for *M. aeruginosa* cultures. As the ambient nitrate: SRP ratio fluctuated, the *M. aeruginosa* C: N ratio was ∼6 from Day 0 to Day 7 and increased to ∼15 on Day 14 (Figure 4C). Piecewise linear regression identified an ambient nitrate: SRP ratio of 5.90 [95% confidence range: (1.51, 10.28)] as the breakpoint that separated different rates of change in C: N ratios for *M. aeruginosa* (Supplementary Table 2). In contrast, the ambient nitrate: SRP ratio of *D. flos-aquae* cultures varied from 17 to 14 before Day 9 and decreased to less than 0.05 on Day 14, and the *D. flos-aquae* C: N ratio was less than 6 for 11 days and slightly increased to ∼8 on Day 14 (Figure 4D). Piecewise linear regression identified an ambient nitrate: SRP ratio of 0.15 [95% confidence range: (0.01, 7.48)] as the breakpoint for *D. flos-aquae* under 2,576 μg/L initial N condition (Supplementary Table 2). The initial nitrate-N: SRP ratio was 100 for high-N treatment (16,128 μg/L) and increased rapidly due to SRP depletion. We identified significant linear correlation between cyanobacteria C: N and the ambient nitrate: SRP ratio for *M. aeruginosa* cultures (Supplementary Table 2 and Figure 4E), while *D. flos-aquae* C: N was more constant during the experiment (linear regression, F = 0.0001, *p* = 0.99, df = 26, Figure 4F).

**FIGURE 5:**
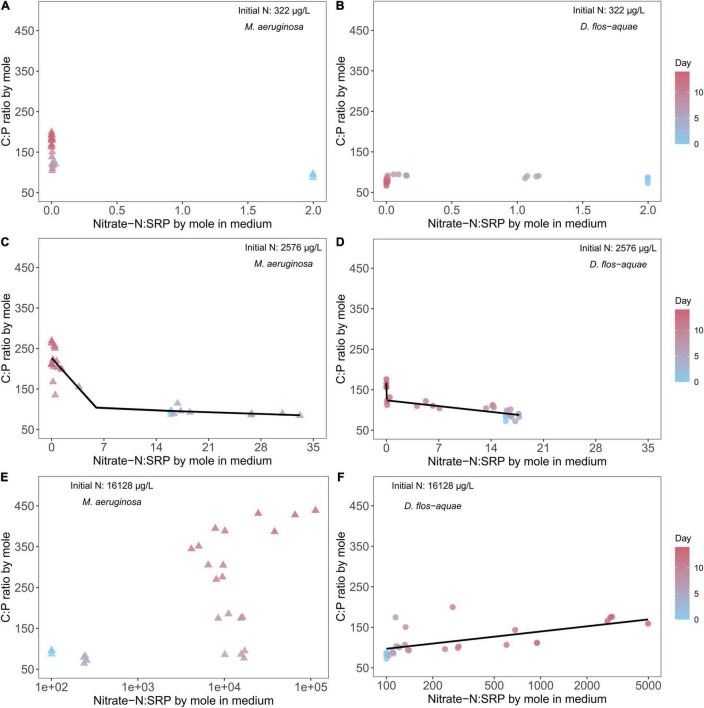
Relationship between cyanobacteria C: P ratio and ambient N: P ratio for *M. aeruginosa* **(A,C,E)** and *D. flos-aquae* **(B,D,F)** under different initial N concentrations.

Under 322 μg/L initial N conditions, we found no correlation between ambient nitrate: SRP ratio and cyanobacteria C: P stoichiometry for two species (Figures 5A,B). In addition, the C: P ratio in *M. aeruginosa* increased from ∼90 to ∼180 as population increased (Figure 5A), and the ambient nitrate: SRP ratio was less than 0.1 after Day 5 during the experiment. On the contrary, the *D. flos-aquae* cultures had a more constrained C: P ratio, and the ambient nitrate: SRP ratio decreased gradually (Figure 5B). Under 2,576 μg/L initial N conditions, we found that cyanobacteria C: P ratios were negatively correlated with ambient nitrate: SRP ratios and we fit piecewise linear regression models to *M. aeruginosa* and *D. flos-aquae* data and identified different breakpoints that separated different changes in rate between species (Supplementary Table 3). For *M. aeruginosa*, the C: P ratio slightly decreased as the ambient nitrate: SRP ratio increased to ∼30, and when the ambient nitrate: SRP was less than 6.07 [breakpoint from piecewise linear regression, 95% confidence range: (3.21, 9.78)], C: P increased to ∼240 at the end of the experiment (Supplementary Table 3 and Figure 5C). In contrast, the *D. flos-aquae* ambient nitrate: SRP ratio only increased to ∼18 on Day 5 and then decreased to below 0.1 on Day 14 when the C: P ratio in *D. flos-aquae* increased from ∼80 to ∼160 (Figure 5D). Under 16,128 μg/L initial N conditions, the C: P ratio of *M. aeruginosa* slightly decreased on Day 5, and from Day 7 to Day 14 the ambient nitrate: SRP ratio increased rapidly to ∼100,000 while C: P ratio increased to ∼ 450 on Day 14 (Figure 5E). For *D. flos-aquae*, the ambient nitrate: SRP ratio was less variable, and we found that C: P ratios increased linearly with ambient nitrate: SRP ratios (Supplementary Table 3 and Figure 5F).

### Cyanobacteria Growth Rate, Phycobilin Pigments, and C:N:P Stoichiometry

**FIGURE 6:**
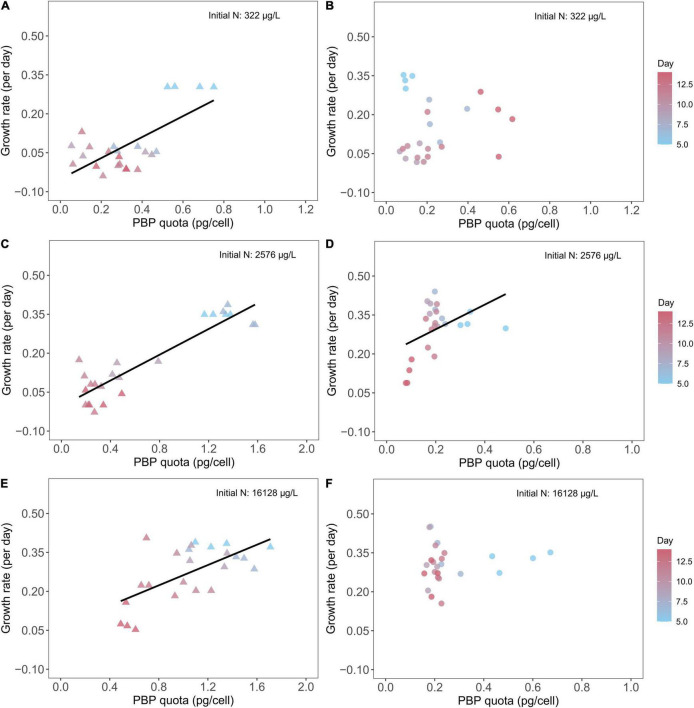
Relationship between cyanobacteria growth rates and PBP cell quota for *M. aeruginosa* **(A,C,E)** and *D. flos-aquae* **(B,D,F)** under different initial N concentrations.

We found *M. aeruginosa* growth rates closely correlated with PBP quota in batch cultures with three different initial nitrate concentrations (Figures 6A,C,E). Overall, *Microcystis* growth rate declined over time with the greatest growth rate on Day 5 and the lowest growth rate on Day 14. Accordingly, we found the PBP quotas varied within a batch culture experiment. With 322 μg/L initial N concentration, *Microcystis* PBP content varied from ∼0.7 pg/cell on Day 5 to less than 0.4 pg/cell on Day 14 (Figure 6A), and the PBP content was promoted by initial N concentration as we saw a PBP content of ∼1.4 pg/cell on Day 5 in *Microcystis* batch cultures with 2,576 μg/L and 16,128 μg/L initial N concentrations (Figures 6C,E). Further, we identified statistically same regression slope between growth rate and PBP cell quota in *Microcystis* cultures with 2,576 and 16,128 μg/L initial N concentrations (Supplementary Table 4). On the contrary, we found no significant correlation between *D. flos-aquae* growth rates and PBP quotas in batch cultures with 322 μg/L (Figure 6B) and 16,128 μg/L (Figure 6F) initial nitrate concentrations. In addition, we found the PBP content in *Dolichospermum* varied from ∼0.1 pg/cell to ∼0.6 pg/cell, regardless of the initial N concentrations in batch cultures.

**FIGURE 7:**
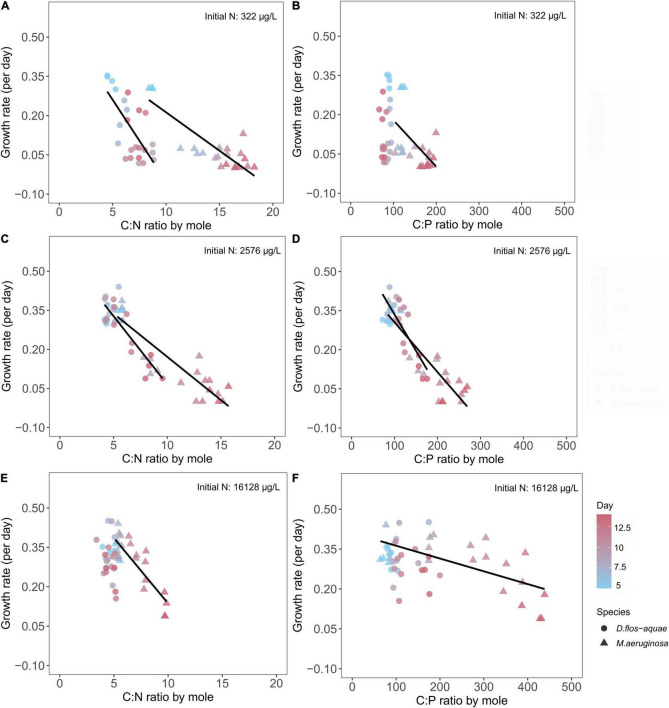
Relationship between cyanobacteria growth rates and C: N ratio **(A,C,E)** and C: P ratios **(B,D,F)** for *M. aeruginosa* and *D. flos-aquae* under different initial N concentrations.

We found negative correlations between growth rate and C: N for *M. aeruginosa* in all three initial N conditions (Figures 7A,C,E). For *D. flos-aquae*, the trend was similar though with different linear regression slopes for the same initial N concentration (Supplementary Table 5). The one exception was *D. flos-aquae* grown with the greatest initial N condition, where we found no correlation between growth rates and C: N ratios (linear regression, F = 1.73, *p* = 0.20, df = 22).

Similarly, we found growth rate decreased linearly with increasing C: P ratios for *M. aeruginosa* in all three initial N conditions (Figures 7B,D,F). The growth rate for *D. flos-aquae*, however, appeared to be independent of C: P ratio under 322 (linear regression, F = 1.97, *p* = 0.17, df = 22) and 16,128 μg/L initial N conditions (linear regression, F = 0.24, *p* = 0.63, df = 22). Furthermore, the growth rates were regulated differently according to C: P ratios between species, as we identified different slopes from linear regressions within a same initial N concentration (Supplementary Table 6).

## Discussion

Nitrogen-rich macromolecules may play a major role in the variability of phytoplankton N stoichiometry because they can represent large fractions of cellular N and their utilization may differ among phytoplankton taxa (Geider and La Roche, 2002). More importantly, some N-rich macromolecules may have the potential to help phytoplankton maintain growth under N-limited conditions, especially in systems where environmental nutrient concentrations fluctuate rapidly (Grover, 2011). Building on our previous work showing the relationship between light and N availability and PBP production by *M. aeruginosa* and *D. flos-aquae* (Wang et al., 2021), we further demonstrated the dynamic phycobilisome metabolism in batch cultures of non-diazotrophic and diazotrophic cyanobacteria. We found that regardless of initial N conditions, *D. flos-aquae* exhibited more constrained C: N and C: P stoichiometry than *M. aeruginosa* as population increased. We also found that ambient N and P concentrations influenced cellular N allocation into PBPs differently for *M. aeruginosa* and *D. flos-aquae*, which can help explain the difference in C: N stoichiometry between two studied species. Our results suggest distinct N metabolism and storage strategies between two globally distributed HAB-forming cyanobacteria species and provide macromolecular basis to quantify and predict aquatic biogeochemical processes.

### Cyanobacteria Nutrient Limitation and Metabolic Traits

We observed different timing in the depletion of dissolved nutrients during batch culture growth experiments with *M. aeruginosa* and *D. flos-aquae*, likely due to interspecific differences in nutrient uptake rates (Figure 1). *Microcystis* is known to uptake P in excess relative to immediate metabolic demand (Jacobson and Halman, 1982), and a more recent study has demonstrated that *Microcystis* N uptake rate was actively regulated according to dissolved N concentrations (Harke and Gobler, 2015). Our results indicated that *Microcystis* may exhibit a more rapid nitrate-N uptake rate than *Dolichospermum;* thus, *Microcystis* depleted the dissolved N pool at an earlier stage as population increased (Figures 1C,D). In addition, cyanobacteria N and P metabolism is biochemically linked, as the P-assimilation genes were upregulated in P-limited cyanobacteria populations when the environmental N pool was enriched (Wang et al., 2018). We found that *Microcystis* batch cultures can deplete environmental P more rapidly than *Dolichospermum* under N-rich conditions, suggesting that cyanobacteria blooms may facilitate the shift from a high-P to a low-P state of a lake differently depending on the species present, and cyanobacteria further mediate nutrient cycling and ecosystem resilience.

Besides nutrient uptake, we found different PBP-N allocations between *Microcystis* and *Dolichospermum*. *Microcystis* regulated PBP-N cell quota dynamically according to ambient nitrate concentrations and largely allocated N in PBP when growing in nutrient-sufficient conditions (Figure 2). However, the PBP production in *Dolichospermum* was less sensitive to nitrate concentration presumably due to the potential for PBP production concomitant with N_2_ fixation (Wang et al., 2021). Ambient SRP concentration may affect cyanobacteria N allocation and phycobilisome metabolism, mainly because P-limited cyanobacteria must reduce light-harvesting pigment inventory to avoid photo-damage (Schwarz and Forchhammer, 2005). The differing N allocation patterns could also arise from different nutrient utilization strategies. Phycobilisome may be a preferred N-storage pool for *Microcystis*, while *Dolichospermum* tend to overcome N limitation by fixing N_2_ as we found PBP cell quota closely associated with *M. aeruginosa* growth rate but not in *D. flos-aquae* cultures (Figure 6). Particularly in batch cultures with low initial nitrate concentrations, we saw *Microcystis* cell concentration continued to increase when the nitrate concentration was depleted. In addition, a previous study suggested that cyanobacteria growing diazotrophically synthesize cyanophycin as a storage of fixed N (Li et al., 2001), and no studies to our knowledge have compared PBP and cyanophycin production in diazotrophic cyanobacteria under variable N availability. A preference for nutrient storage molecules may impact a species’ growth and, more importantly, the nutrient stoichiometry because different macromolecules differ in element composition (Geider and La Roche, 2002).

### Dynamic Changes in Cyanobacteria Nutrient Stoichiometry During Population Growth

The variability of an organism’s element stoichiometry reflects the outcome of many underlying physiological and biochemical adjustments in response to a changing environment (Elser et al., 2000). In this study, the depletion of nitrate and SRP concentrations resulted in temporally varied ambient nitrate: SRP ratios, which substantially altered cyanobacteria C: N and C: P ratios. Consistent with our hypothesis, we found that *D. flos-aquae* populations were more constrained in C: N stoichiometry than *M. aeruginosa*, regardless of the initial N concentration of a batch culture (Figure 4). Our results added more evidence indicating that diazotrophic cyanobacteria have less variable C: N stoichiometry (Supplementary Figure 5; Osburn et al., 2021). Although regression curves appeared similar, we found distinct difference in C: N stoichiometry variabilities in *Microcystis* and *Dolichospermum*. The metabolism of phycobilisome may help explain the interspecific variations in C: N stoichiometry as we found close negative correlation between *Microcystis* PBP quota and C: N ratios but not in *Dolichospermum* cultures (Supplementary Figure 6). *Microcystis* may re-direct C metabolism toward glycogen accumulation in response to N starvation, which causes an increase in C: N ratios (Forchhammer and Selim, 2020). In addition, elevated C: N ratio in *Microcystis* may be attributed to the formation of C-rich polysaccharide under N-limited conditions (Duan et al., 2021), thereby cells may cluster together to form colonies. Colonial *Microcystis* blooms are commonly found in field studies, and a series of abiotic and biotic factors may induce colony formation (Xiao et al., 2018), which in turn provides *Microcystis* spp. many ecological protection from environmental stressors. Although we found increased C quota in N-limited *M. aeruginosa* (Supplementary Figure 4), we did not observe the colony formation in our cultures (Supplementary Figure 7). In contrast, N-rich *D. flos-aquae* cultures maintained C: N ratios that were close to the Redfield ratio (Redfield, 1958) as population increased (Figure 7E), consistent with early studies on other phytoplankton groups (Goldman et al., 1979).

The interspecific differences in C: P stoichiometry may be attributed to P storage flexibilities (Martin et al., 2014). Although the accumulation of inorganic P polymer (polyphosphate) under P-rich conditions is common among cyanobacteria, studies have reported distinct patterns for P storage in different species (Wan et al., 2019) and strains (Willis et al., 2017). According to our observations, *M. aeruginosa* appears to have more flexibility in P quota than *D. flos-aquae* (Supplementary Figure 3). As a result, under P limitation but with sufficient N supply, *M. aeruginosa* were able to expand biomass (Supplementary Figure 4), although with declined growth rate, stretching the C: P ratio up to 400. Comparable findings were reported for other unicellular non-diazotrophic cyanobacteria (Bertilsson et al., 2003). On the contrary, *D. flos-aquae* maintained more constrained C: P ratios during early and late growth stages, suggesting a pronounced interspecific stoichiometric variability for cyanobacteria blooms.

Although the application of GRH on phytoplankton has been questioned (Flynn et al., 2010), we observed the particulate P cell quota increased linearly with the growth rate for *M. aeruginosa* populations under intermediate and high initial N conditions (Supplementary Figures 3B,C), but not for N-limited populations (Supplementary Figure 3). This is likely caused by *M. aeruginosa* growth being primarily limited by N rather than P in our low-N treatment (Figure 1). A similar positive correlation between growth rate and P quota was expected in *Dolichospermum* because the dependence of N_2_ fixation and diazotrophic growth on P availability has been reported in other filamentous N_2_-fixing cyanobacteria (Degerholm et al., 2006). However, we found no correlation between P cell quota and growth rate in *D. flos-aquae* populations (Supplementary Figures 3,E,F). These discrepancies in the relationship between P cell quota and growth rate for both species are likely caused by non-limiting P conditions which are known to decouple the RNA-P-growth rate relationships (Acharya et al., 2004). A negative correlation between growth rate and C: P ratios has been reported in other phytoplankton (Goldman et al., 1979; Hillebrand et al., 2013), and our research supports this strongly for *M. aeruginosa* and weakly for *D. flos-aquae*.

### Conclusion and Implications

Consistent with our hypotheses, we found that *Dolichospermum* exhibited more constrained C: N stoichiometry than *Microcystis* during population increase in batch cultures, which may be attributed to their distinct regulations on phycobilisome metabolism and N allocation. Nitrogen allocation to PBPs depended on ambient nutrient concentration regardless of species, but interspecific eco-physiological trait differences also contributed to PBP regulation. Although batch culture studies may not represent the complex community dynamics of natural systems, our results indicate that differing traits in non-diazotrophs (*M. aeruginosa*) and diazotrophs (*D. flos-aquae*) can determine bloom persistence/magnitude and may even contribute to toxin production (Van de Waal et al., 2014) and ecosystem nutrient cycling (Cottingham et al., 2015). Our study quantitatively assessed the N allocation and elemental stoichiometry spanning a wide range of environmental N concentrations that may fuel cyanobacteria blooms in eutrophic lakes (Scott et al., 2019). Therefore, our study provides a framework that links cyanobacteria stoichiometry to N-rich macromolecule dynamics and illuminates a potential role for these macromolecules in regulating aquatic biogeochemical processes.

## Data Availability Statement

The original contributions presented in the study are included in the article/Supplementary Material, further inquiries can be directed to the corresponding author.

## Author Contributions

JW, JF, and JS conceived the ideas and designed the methodology. JW, NW, and JS performed the experiments, data analysis, and wrote the manuscript. All authors contributed to the analysis and writing of the manuscript.

## Author Disclaimer

The content is solely the responsibility of the authors and does not necessarily represent the official views of the National Institutes of Health.

## Conflict of Interest

The authors declare that the research was conducted in the absence of any commercial or financial relationships that could be construed as a potential conflict of interest.

## Publisher’s Note

All claims expressed in this article are solely those of the authors and do not necessarily represent those of their affiliated organizations, or those of the publisher, the editors and the reviewers. Any product that may be evaluated in this article, or claim that may be made by its manufacturer, is not guaranteed or endorsed by the publisher.
