# Supplementary material for: Dynamic Phycobilin Pigment Variations in Diazotrophic and Non-diazotrophic Cyanobacteria Batch Cultures Under Different Initial Nitrogen Concentrations
Source: Front Microbiol. 2022 Jun 2;13:850997. doi: 10.3389/fmicb.2022.850997 (PMC9201475; doi:10.3389/fmicb.2022.850997)
Supplement: Supplementary file 1 [file Table_1.DOCX]

Supplementary Information

Dynamic phycobilin pigment variations in diazotrophic and non-diazotrophic cyanobacteria batch cultures under different initial nitrogen concentrations

Jingyu Wang^1,2*^, Nicole D. Wagner^2^, James M. Fulton^4^, J. Thad Scott^1,2,3^

^1^The Institute of Ecological, Earth & Environmental Sciences, Baylor University, Texas, USA

^2^Center for Reservoir and Aquatic Systems Research, Baylor University, Waco, Texas, USA

^3^Department of Biology, Baylor University, Waco, Texas, USA

^4^Department of Geosciences, Baylor University, Waco, Texas, USA

*** Correspondence:**Corresponding Author
The Institute of Ecological, Earth & Environmental Sciences, Baylor University, Texas, USA

jingyu_wang1@baylor.edu


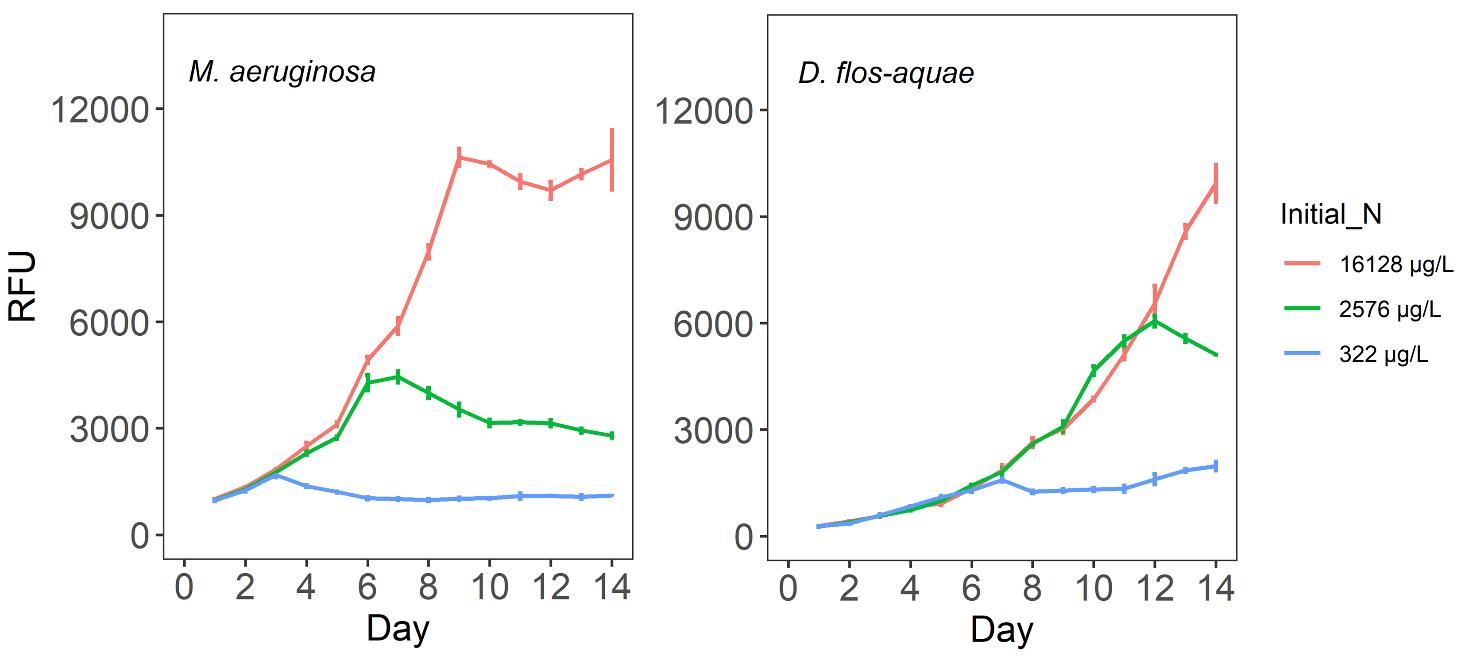


Fig. S1 Temporal variations of *in vivo* chlorophyll-*a* fluorescence for *M. aeruginosa* and *D. flos-aquae* under different initial N concentrations.


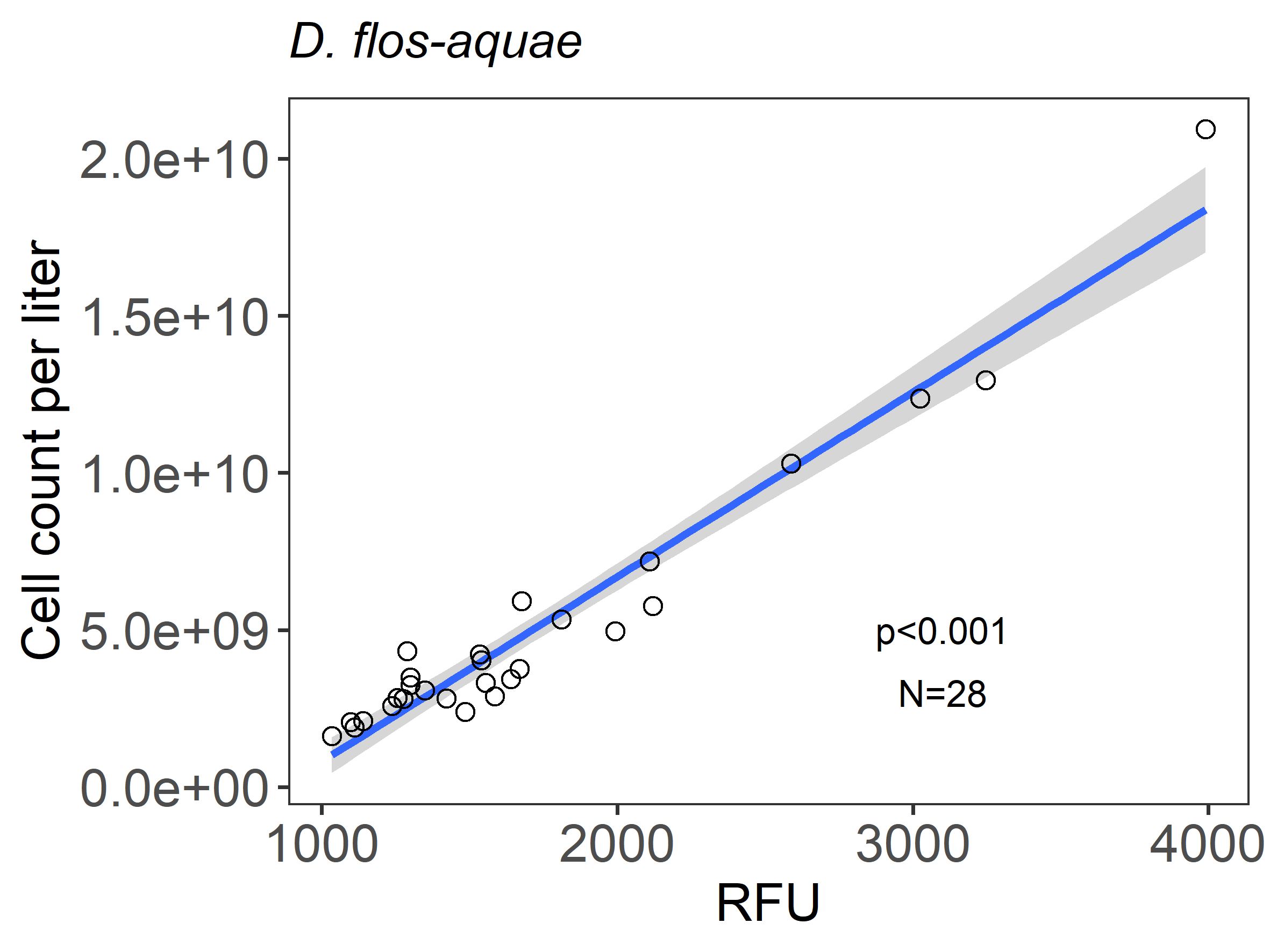


Fig. S2 Correlation between RFU and cell concentrations for *D. flos-aquae*.


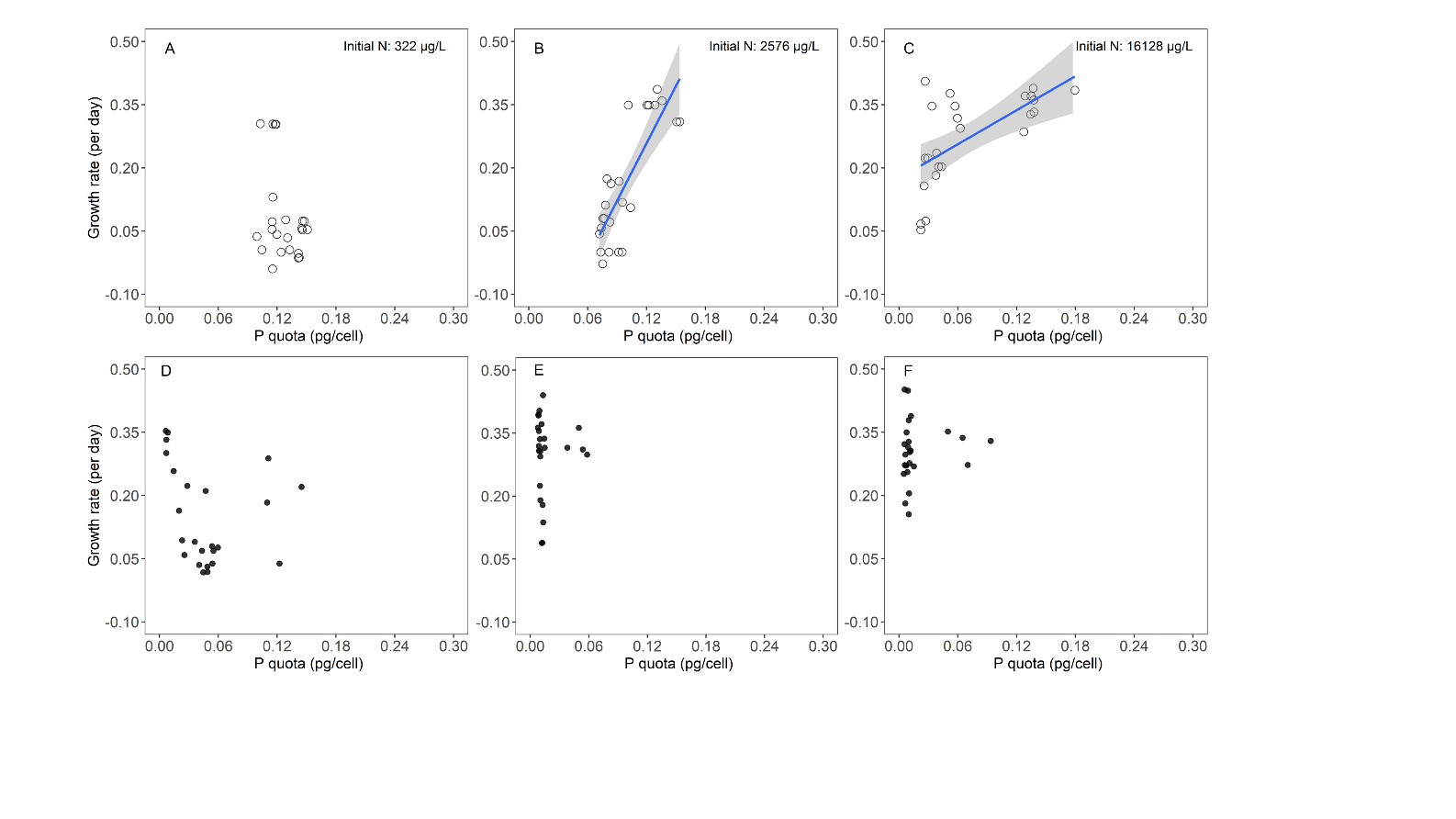


Fig. S3 Growth rate and particulate phosphorus (P) cell quota for *M. aeruginosa* (A: 322 µg/L, B: 2576 µg/L, C: 16128 µg/L) and *D. flos-aquae* (D: 322 µg/L, E: 2576 µg/L, F: 16128 µg/L) under different initial N conditions. Regression line was added when there was a significant slope (*p* <0.05).


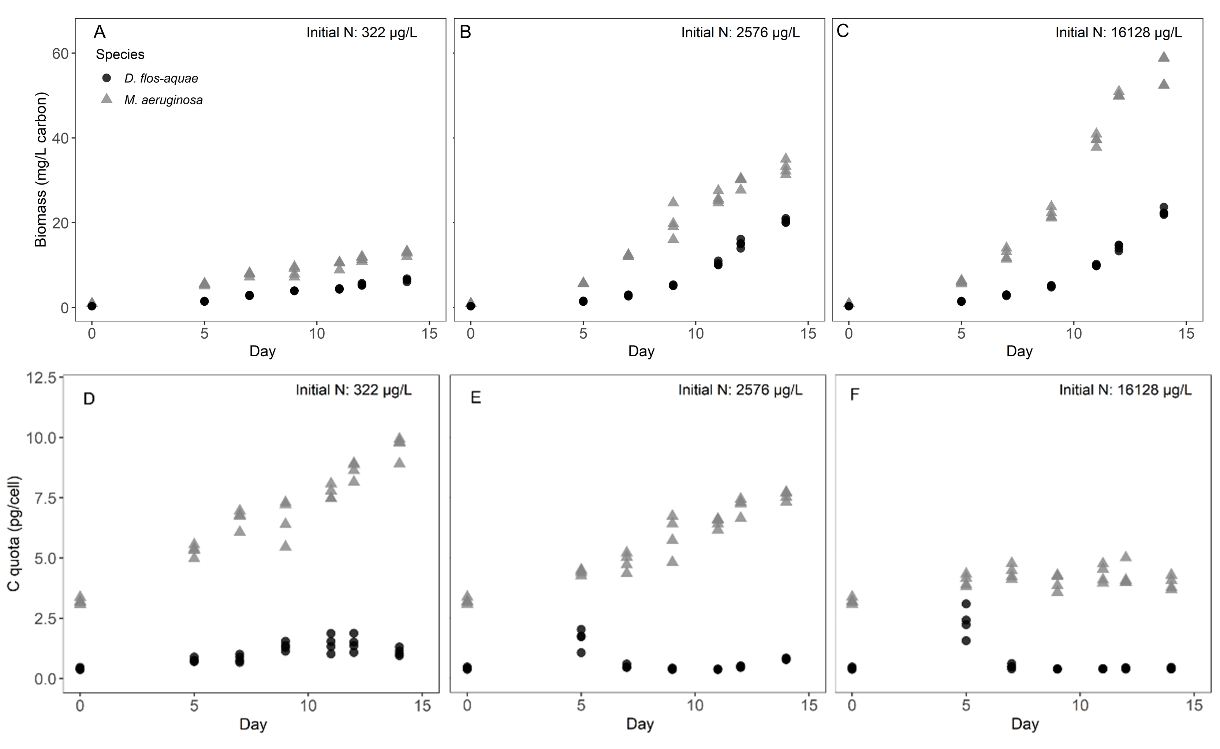


Fig. S4 Temporal variations of biomass (mg/L carbon) and carbon (C) cell quota for *M. aeruginosa* and *D. flos-aquae* with different initial N conditions.


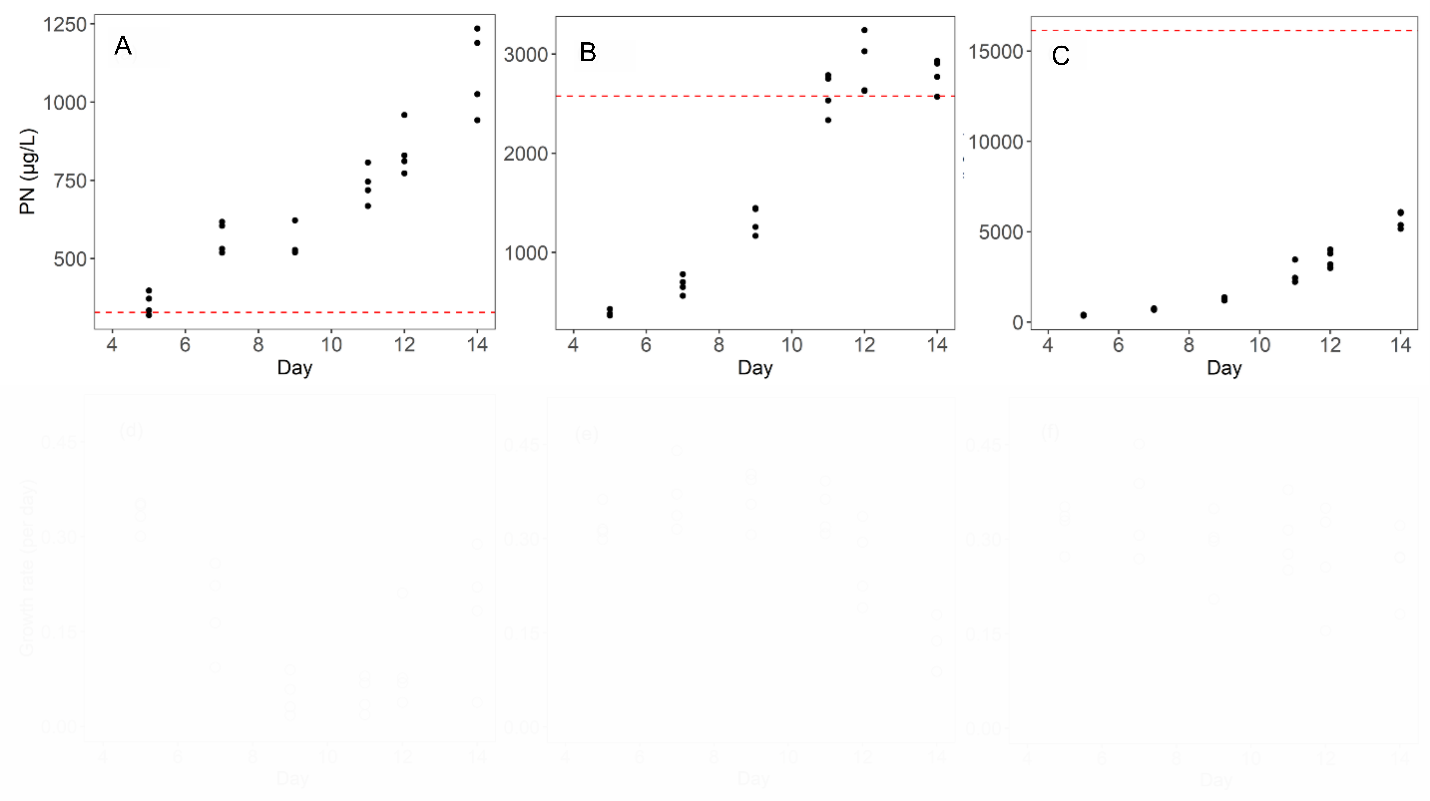
 Fig. S5 Temporal variations of particulate N (PN) concentrations for *D. flos-aquae* with different initial N conditions (A: 322 µg/L, B: 2576 µg/L, C: 16128 µg/L). Red dashed line denotes initial nitrate concentration in the medium, data above the red dashed line indicates N_2_-fixation. Note the difference in Y axis.


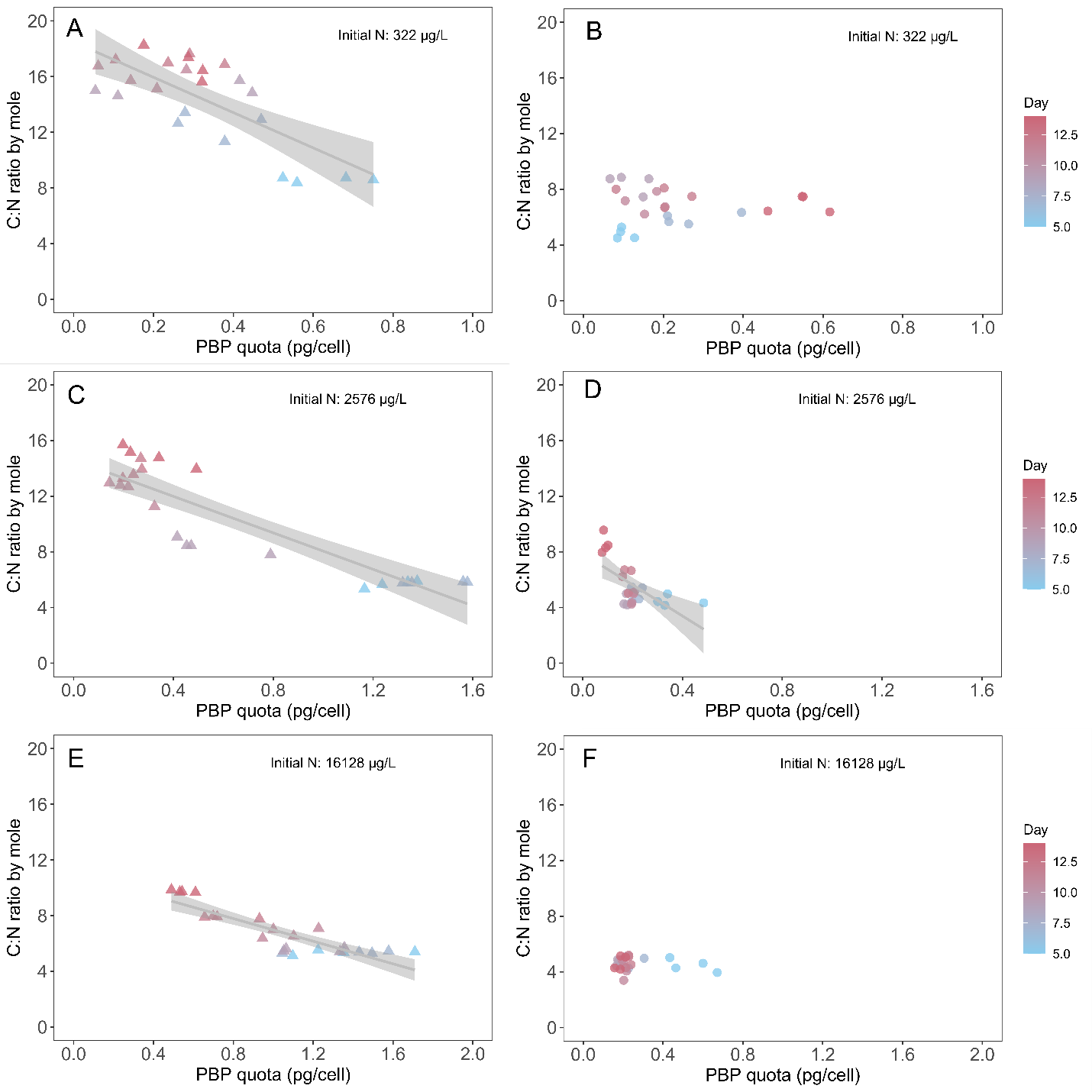


Fig. S6 Correlation between cyanobacteria C: N stoichiometry and PBP cell quota in *M. aeruginosa* (A, C, E) and *D. flos-aquae* (D, E, F).


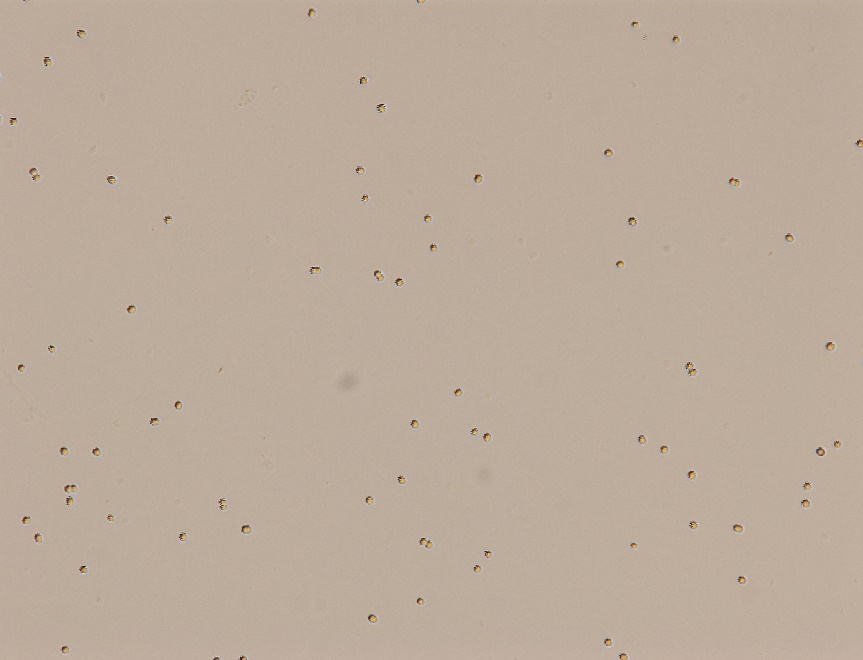


Fig. S7 Microscope image of *Microcystis aeruginosa* culture in this study.

Table S1 Regression equations between SRP concentration and PBP-N cell quota for *M. aeruginosa* and *D. flos-aquae* under different initial N concentrations

| Species | Initial N concentration | | Equation | R^2^ | *p* value |
| --- | --- | --- | --- | --- | --- |
| *M. aeruginosa* | 322 µg/L | Y1= 8.83E$-$06x+0.0239  Y2= 6.15E$-$04x$-$0.0982 | | 0.867 | <0.001 |
| *M. aeruginosa* | 2576 µg/L | Y1= 0.0044x+0.0149  Y2=$-$7.03E-05x+0.141 | | 0.918 | <0.001 |
| *M. aeruginosa* | 16128 µg/L | Y=0.12 x/ (0.36 + x) | | 0.440 | 0.0142 |
| *D. flos-aquae* | 322 µg/L | Y1=$-$5.08E-04x+0.081  Y2= 1.32 E-06x+0.0016 | | 0.747 | <0.001 |
| *D. flos-aquae* | 2576 µg/L | Y= 4.47 E-06x+0.0131 | | 0.161 | 0.019 |
| *D. flos-aquae* | 16128 µg/L | ns | | 0.058 | 0.115 |

Table S2 Equation obtained from piecewise linear regression between nitrate-N: SRP ratio and cyanobacteria C: N stoichiometry under different initial N concentrations

| Species | Initial N concentration | | Equation 1 | Equation 2 | Breakpoint |  | *p* value |
| --- | --- | --- | --- | --- | --- | --- | --- |
| *M. aeruginosa* | 322 µg/L | na | | na | na |  | ns |
| *M. aeruginosa* | 2576 µg/L | Y= $-$1.402x+13.286 | | Y=0.088x+4.513 | 5.90 |  | <0.001 |
| *M. aeruginosa* | 16128 µg/L | Y= 4.540E-05x+5.86 | | na | na |  | <0.001 |
| *D. flos-aquae* | 322 µg/L | Y= $-$10.86x+2.64 | | Y= $-$0.0088x+4.81 | 0.24 |  | <0.001 |
| *D. flos-aquae* | 2576 µg/L | Y= $-$12.56x+9.44 | | Y=0.022x+5.06 | 0.15 |  | <0.001 |
| *D. flos-aquae* | 16128 µg/L | na | | na | na |  | 0.99 |

Table S3 Equation obtained from piecewise linear regression between nitrate-N: SRP ratio and cyanobacteria C: P stoichiometry under different initial N concentrations

| Species | Initial N concentration | | Equation 1 | Equation 2 | Breakpoint |  | *p* value |
| --- | --- | --- | --- | --- | --- | --- | --- |
| *M. aeruginosa* | 322 µg/L | ns | | ns | ns |  | ns |
| *M. aeruginosa* | 2576 µg/L | Y= $-$20.93x+244.41 | | Y=$-$0.613x+104.98 | 6.07 |  | <0.001 |
| *M. aeruginosa* | 16128 µg/L | ns | | ns | ns |  | ns |
| *D. flos-aquae* | 322 µg/L | ns | | ns | ns |  | 0.41 |
| *D. flos-aquae* | 2576 µg/L | Y= $-$403.6x+95.01 | | Y= $-$2.05x+124.01 | 0.004 |  | <0.001 |
| *D. flos-aquae* | 16128 µg/L | Y= 1.783E-02x+103.4 | | ns | ns |  | <0.001 |

Table S4 Summary of *p* values from post-hoc multiple comparison of slopes of linear regression for growth rate and PBP cell quota among groups using SMATR

| Group | *Dolichospermum*-high | *Microcystis*-high | *Dolichospermum* -low | *Microcystis* -low | *Dolichospermum* -intermediate | *Microcystis* -intermediate |
| --- | --- | --- | --- | --- | --- | --- |
| *Dolichospermum*-high | 1.0 | 0.201 | 0.001 | 0.001 | 0.024 | 0.303 |
| *Microcystis*-high | 0.201 | 1.0 | 0.001 | 0.001 | 0.198 | 0.524 |
| *Dolichospermum* -low | 0.001 | 0.001 | 1.0 | 0.002 | 0.001 | 0.001 |
| *Microcystis* -low | 0.001 | 0.001 | 0.002 | 1.0 | 0.064 | 0.001 |
| *Dolichospermum* -intermediate | 0.024 | 0.198 | 0.001 | 0.064 | 1.0 | 0.063 |
| *Microcystis* -intermediate | 0.303 | 0.524 | 0.001 | 0.001 | 0.063 | 1.0 |

Table S5 Summary of *p* values from post-hoc multiple comparison of slopes of linear regression for growth rate and C: N ratio among groups using SMATR

| Group | *Dolichospermum*-high | *Microcystis*-high | *Dolichospermum* -low | *Microcystis* -low | *Dolichospermum* -intermediate | *Microcystis* -intermediate |
| --- | --- | --- | --- | --- | --- | --- |
| *Dolichospermum*-high | 1.0 | 0.003 | 0.034 | 0.001 | 0.001 | 0.001 |
| *Microcystis*-high | 0.003 | 1.0 | 0.082 | 0.002 | 0.720 | 0.003 |
| *Dolichospermum* -low | 0.034 | 0.082 | 1.0 | 0.001 | 0.089 | 0.001 |
| *Microcystis* -low | 0.001 | 0.002 | 0.001 | 1.0 | 0.003 | 0.924 |
| *Dolichospermum* -intermediate | 0.001 | 0.720 | 0.089 | 0.003 | 1.0 | 0.002 |
| *Microcystis* -intermediate | 0.001 | 0.003 | 0.001 | 0.924 | 0.002 | 1.0 |

Table S6 Summary of *p* values from post-hoc multiple comparison of slopes of linear regression for growth rate and C: P ratio among groups using SMATR

| Group | *Dolichospermum*-high | *Microcystis*-high | *Dolichospermum* -low | *Microcystis* -low | *Dolichospermum* -intermediate | *Microcystis* -intermediate |
| --- | --- | --- | --- | --- | --- | --- |
| *Dolichospermum*-high | 1.0 | 0.01 | 0.001 | 0.038 | 0.022 | 0.786 |
| *Microcystis*-high | 0.001 | 1.0 | 0.001 | 0.001 | 0.001 | 0.001 |
| *Dolichospermum* -low | 0.001 | 0.001 | 1.0 | 0.001 | 0.001 | 0.001 |
| *Microcystis* -low | 0.038 | 0.001 | 0.001 | 1.0 | 0.856 | 0.003 |
| *Dolichospermum* -intermediate | 0.022 | 0.001 | 0.001 | 0.856 | 1.0 | 0.003 |
| *Microcystis* -intermediate | 0.786 | 0.001 | 0.001 | 0.003 | 0.003 | 1.0 |
